# Simultaneous high-efficiency base editing and reprogramming of patient fibroblasts

**DOI:** 10.1016/j.stemcr.2021.10.017

**Published:** 2021-11-24

**Authors:** Sami Jalil, Timo Keskinen, Rocío Maldonado, Joonas Sokka, Ras Trokovic, Timo Otonkoski, Kirmo Wartiovaara

**Affiliations:** 1 Stem Cells and Metabolism Research Program, Faculty of Medicine, University of Helsinki, 00290 Helsinki, Uusimaa, Finland; 2 Department of Pediatrics, Helsinki University Hospital, 00290 Helsinki, Uusimaa, Finland; 3 Department of Clinical Genetics, Helsinki University Hospital, 00290 Helsinki, Uusimaa, Finland

**Keywords:** CRISPR-Cas9, gene editing, disease modeling, reprogramming, induced pluripotent stem cells, CADASIL, familial hypercholesterolemia, NOTCH3, LDLR, Finnish-founder mutation

## Abstract

Human induced pluripotent stem cells (hiPSCs) allow *in vitro* study of genetic diseases and hold potential for personalized stem cell therapy. Gene editing, precisely modifying specifically targeted loci, represents a valuable tool for different hiPSC applications. This is especially useful in monogenic diseases to dissect the function of unknown mutations or to create genetically corrected, patient-derived hiPSCs. Here we describe a highly efficient method for simultaneous base editing and reprogramming of fibroblasts employing a CRISPR-Cas9 adenine base editor. As a proof of concept, we apply this approach to generate gene-edited hiPSCs from skin biopsies of four patients carrying a Finnish-founder pathogenic point mutation in either *NOTCH3* or *LDLR* genes. We also show LDLR activity restoration after the gene correction. Overall, this method yields tens of gene-edited hiPSC monoclonal lines with unprecedented efficiency and robustness while considerably reducing the cell culture time and thus the risk for *in vitro* alterations.

## Highlights

• Simultaneous base editing and reprogramming efficiently generates corrected hiPSCs
• RNA-delivered ABE edits the targeted loci without DSBs or detectable off-targets
• The base-edited hiPSCs exhibit normal karyotype and pluripotency
• Differentiated patient hiPSCs show phenotype restoration upon gene correction

## Graphical abstract

**Figure:**
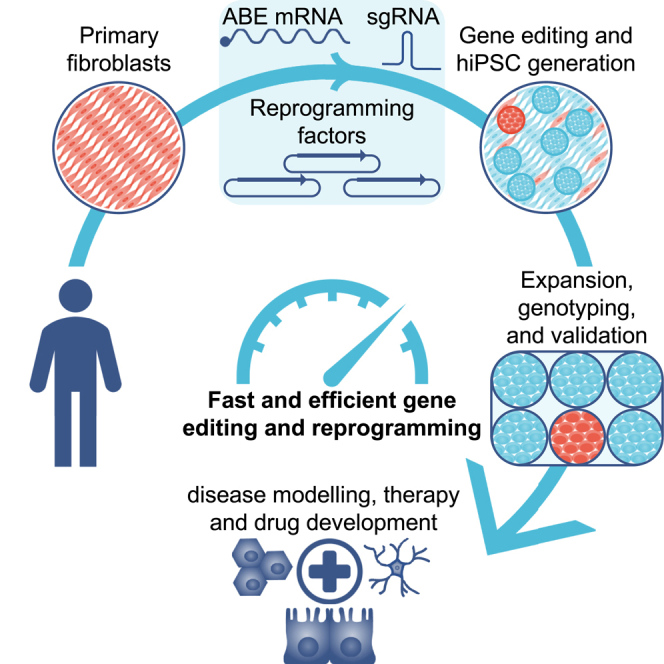

## Teaser

In this study, Jalil and colleagues develop and test a method for simultaneous hiPSC reprogramming and RNA-delivered adenine base editing. Starting from primary patient-derived fibroblasts, this approach yields several on-target edited isogenic hiPSC lines, with remarkable speed and editing efficiency and without compromising their genetic integrity or their pluripotency quality. The gene editing corrects the cellular disease phenotype.

## Introduction

Human induced pluripotent stem cells (hiPSCs) represent an invaluable tool for developmental biology, regenerative and genetic medicine, therapeutic-target discovery, and drug screening. Since the first reports illustrating the derivation of hiPSCs from somatic cells (Takahashi et al., 2007; Yu et al., 2007), the field has rapidly expanded and holds the potential to deliver off-the-shelf cell therapies (Crow, 2019). Key advantages of hiPSC derivation are the simplicity and the efficacy of the reprogramming technique: the transient expression of defined reprogramming factors, such as OCT4, SOX2, KLF4, and MYC, suffices to generate pluripotent cells from a small sample of human somatic tissue. hiPSCs maintain their pluripotency even throughout extensive culture and, under the right stimuli, differentiate into virtually any cell type of the human body. Consequently, they are widely employed in establishing cell and organoid models for inherited diseases.

Human embryonic stem cells (hESCs) have been successfully employed in several clinical trials (Mehat et al., 2018; Menasché et al., 2018; Schwartz et al., 2015) (Viacyte, https://clinicaltrials.gov: NCT03163511, and Asterias Biotherapeutics, https://clinicaltrials.gov: NCT02302157). However, the limited availability of hESCs hinders their application as a therapeutic option. Their proliferation capacity, pluripotency, and virtually unlimited supply make hiPSCs strong candidates to replace hESCs in autologous cell transplantation therapy and in tissue engineering and this has already succeeded in a clinical trial (Mandai et al., 2017).

Genome editing techniques enable the correction of pathogenic mutations in patient-derived hiPSCs or the introduction of the desired mutation into a control hiPSC line. By comparing differentiated hiPSC lines with identical genetic backgrounds but differing in the edited genomic regions, researchers can link a particular genotype to its phenotype in specific cell types. Genome editing techniques relying on double-strand breaks (DSBs) to induce homology-directed repair (HDR) are challenging in hiPSCs since these cells are more likely to resolve DSBs by the error-prone non-homologous end joining (NHEJ) instead of HDR (Guo et al., 2018). Moreover, DSBs frequently result in unwanted DNA insertions or deletions (indels), chromosomal aberrations, apoptosis, or population enrichment in oncogenic mutations (Chapman et al., 2012; Haapaniemi et al., 2018; Ihry et al., 2018). In contrast, the adenine base editor (ABE) (Gaudelli et al., 2017), which converts a targeted A⋅T base pair to a G⋅C base pair, does not induce DSBs and therefore limits the generation of undesired mutations or chromosomal aberrations (Komor et al., 2018). Moreover, the ABE presents a lower off-target than that observed with canonical SpCas9 (Jin et al., 2019; Komor et al., 2018; Liang et al., 2019).

Here, we developed an efficient RNA-based delivery system for A⋅T to G⋅C conversion in patient-derived primary fibroblasts employing the ABEmax (Koblan et al., 2018), a more efficient and codon-optimized ABE version. We combined this approach with episomal vector reprogramming, creating a fast and robust method for simultaneous derivation of hiPSC lines and specific point mutation correction in a single straightforward procedure. Starting from a patient skin biopsy, this approach quickly yields tens of genetically corrected hiPSC monoclonal lines with an efficiency consistently above 96%. Previous studies employing SpCas9 (Howden et al., 2015; Howden et al., 2018; Kim et al., 2016; Tidball et al., 2017, 2018; Wen et al., 2018) achieved simultaneous reprogramming and DSB-dependent gene editing, which is particularly powerful in generating knockouts. By replacing SpCas9 with the ABEmax and improving the delivery efficiency, the simple DBS-free method presented in this article significantly improves the implementation and efficacy of base editing in hiPSC reprogramming and facilitates their use for research, biobanking, and future therapeutic applications.

## Results

### Highly efficient RNA-delivered base editing and simultaneous hiPSC generation

**Figure 1:**
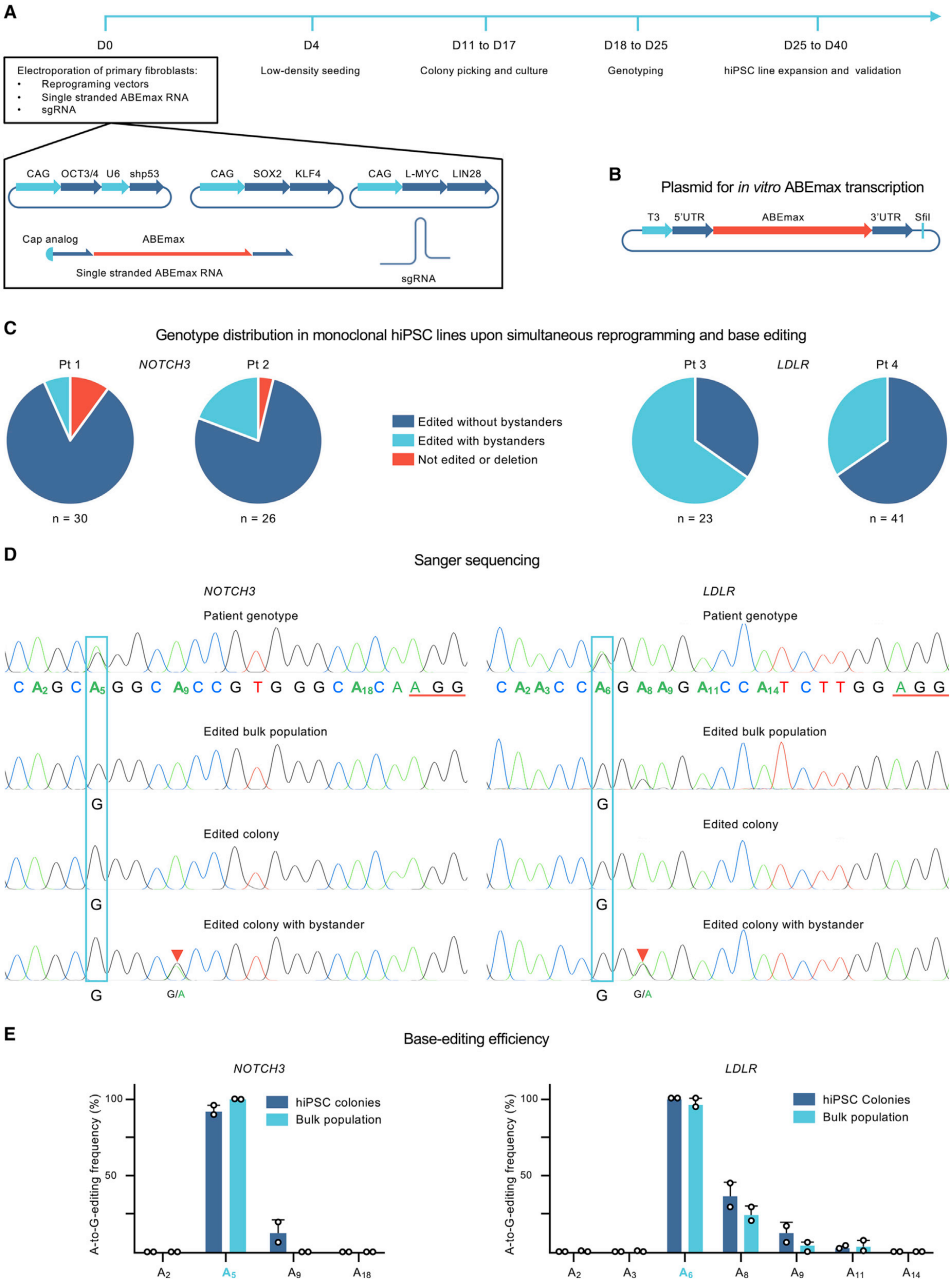
Highly efficient base editing and simultaneous hiPSC generation (A) Workflow for simultaneous base editing and reprogramming. Patient-derived primary fibroblasts are electroporated with three reprogramming plasmids plus an RNA construct encoding the ABEmax base editor and a sgRNA. Four days after electroporation, the cells are split and seeded in low density. From day 11 to day 17, the hiPSC monoclonal colonies are visible and ready to be transferred to a different plate for expansion. Between days 18 and 25, the hiPSCs undergo expansion and Sanger sequencing. The monoclonal hiPSC lines with the desired phenotype are then validated. (B) Plasmid employed as a template for *in vitro* RNA transcription. The T3 RNA polymerase binds the T3 promotor region and transcribes the ABEmax sequence flanked by 5′ and 3′ UTRs. The transcription ends at the SfiI restriction site that was previously digested with this endonuclease. The T3 RNA polymerase simply cannot continue transcribing beyond the restricted end. (C) Distribution of genotypes in clonal base-edited hiPSC lines derived from four independent heterozygote patients: two patients, Pt 1 and Pt 2, carrying the mutation in *NOTCH3* and two with the mutation in *LDLR*, Pt 3 and Pt 4. One hundred twenty monoclonal colonies were sequenced in total: 30, 26, 23, and 41, respectively. (D) Sanger sequences in 5′-to-3′ orientation illustrating the observed genotypes. The orange underlined bases (AGG) are the PAM region, whereas the 20 remaining bases are the sgRNA sequence. A light blue box marks the targeted adenine. The orange triangles point to examples of bystander adenine editing. (E) Bar graphs depicting the editing efficiency for the targeted and the bystander adenines in the window of editing. The targeted adenine is marked in light blue. The dark blue bars summarize the mean A-to-G editing frequency observed when combining the individual genotypes of each monoclonal colony derived from the four independent patients: *NOTCH3* (Pt 1, n = 30; Pt 2, n = 26) and *LDLR* (Pt 3, n = 23; Pt 4, n = 41). The light blue bars summarize the mean A-to-G editing frequency observed in the independently edited bulk populations after simultaneous reprogramming and base editing, *NOTCH3* (n = 2, Pt 1 and Pt 2) and *LDLR* (n = 2, Pt 3 and Pt 4). The error bars show the standard deviation.

Converting A⋅T base pairs to G⋅C base pairs has the potential to correct 48% of all the described pathogenic human single-nucleotide polymorphisms (SNPs) without introducing DSBs (Gaudelli et al., 2017). In this study, we developed a straightforward method that allows clean A⋅T to G⋅C conversion and fast generation of isogenic hiPSC lines by simultaneous delivery of an ABE as mRNA and episomal reprogramming factors (Figure 1A). To guarantee the high and transient expression of the ABEmax and to minimize DNA toxicity, we designed an *in vitro* transcribed RNA construct mimicking the structure of human mRNA (Figure 1A). The DNA template employed for RNA transcription contains the original ABEmax sequence inside an optimized backbone for *in vitro* transcription (IVT) (Torres-Padilla et al., 2007) (Figure 1B). The UTRs in this construct stabilize the RNA to improve its translation potential. In addition, the 7-methyl guanosine cap at 5′ mimics the structure of human mRNAs found *in vivo*. For hiPSC induction, we employed episomal vector reprogramming (Okita et al., 2011).

We applied this approach to correct two different Finnish-founder pathogenic point mutations and simultaneously generated hiPSCs. We employed primary fibroblasts from four independent skin biopsies. Patients 1 and 2 were heterozygous for a c.475C > T transition in exon 3 of *NOTCH3* (Figure 1C), a dominant mutation causing CADASIL (Mykkänen et al., 2004). Patients 3 and 4 presented a heterozygous c.1784G > A transition in exon 12 of *LDLR* (Figure 1C), causing a dominant form of familial hypercholesterolemia (FH-Pogosta) (Vuorio et al., 2001).

After the electroporation of patient-derived primary fibroblasts with the episomal reprogramming vectors, the mRNA ABEmax construct and the single-guide RNA (sgRNA; which binds to the ABE and defines the genomic target to be modified), our method generated hundreds of monoclonal colonies. Sanger sequencing of the 120 individual colonies showed that 96.7% carried the desired A-to-G edit on the targeted position (Figure 1D): 27 of 30 hiPSC lines from patient 1 and 25 of 26 hiPSC lines from patient 2 presented the A-to-G edition on the targeted *NOTCH3* mutation*.* Meanwhile, all the hiPSC lines from patients 3 and 4 (n = 23 and n = 41, respectively) carried the desired A-to-G edit on the targeted *LDLR* mutation. In summary, just 4 clones of 120 resulted as negative for the desired A⋅T to G⋅C conversion: 1 presented a −1 bp deletion, another a +5 bp insertion, and 2 conserved the original genotype (Figure 1D).

ABEmax is designed to convert any adenine to guanine inside a defined DNA region matching with the sgRNA. This region, called the window of editing, includes bases 4 to 11 when the protospacer-adjacent motif (PAM) (NGG) locates on positions 21-23 (Koblan et al., 2018). As more than one adenine lay inside the window of editing in the loci of interest in these experiments, some of the colonies edited on the targeted pathogenic point mutation also showed bystander edits: collateral A-to-G conversion in nearby adenines (Figures 1D and 1E). Nevertheless, two-thirds (80 of 120) of the sequenced hiPSC lines presented an accurate A-to-G edit solely on the targeted adenine.

To assess the editing efficiency at a cell population level, we collected one-third of all cells 20 days after electroporating primary fibroblasts with the reprogramming vectors, the ABEmax, and the sgRNA. Each of these four bulk population samples, one per patient, contained the DNA of at least 3 million cells. Sanger sequencing of their PCR amplicons revealed an editing pattern analogous to that obtained by analyzing the monoclonal colonies (Figure 1E). The on-target editing efficiencies on *LDLR* in the bulk population derived from patients 3 and 4 were 94% and 100%, respectively, whereas patients 1 and 2 showed an on-target A⋅T to G⋅C conversion efficiency of 100% on *NOTCH3*. Following the canonical window of editing for the ABEmax, the adenines located in positions 2, 3, 14, and 18 were not edited, while we saw significant A⋅T to G⋅C conversion in positions 5, 6, 8, 9, and 11. The editing efficiency peaked at positions 5 and 6 and then decreased toward the PAM (Figure 1E). Thus, starting from primary fibroblast cultures, this method yielded tens of monoclonal hiPSC lines with the desired correction in 5 weeks.

Finally, we established one non-ABE hiPSC line from each of the four patients by episomal vector reprogramming without exposure to the base editor. To test the performance of our ABEmax RNA construct directly in hiPSCs, we electroporated 1 million hiPSCs from each of the four independent lines with the ABEmax RNA and the corresponding sgRNA. Seven days post-electroporation, the Sanger sequencing of the hiPSC bulk populations revealed that the on-target editing efficiency was on average 75.5% for the *NOTCH3* patients 1 and 2 hiPSC lines and was 92.5% for the *LDLR* patients 3 and 4 (Figure S1A). The bystander adenines inside the window of editing displayed lower editing efficiencies, ranging from 0% to 8.5%.

### RNA-delivered ABEmax editing combined with transgenic reprogramming produces high-quality hiPSCs

Once we ensured that our RNA-delivered base-editing system coupled to the episomal vectors for reprogramming worked across different loci, we assessed whether the addition of the ABEmax affected the hiPSC generation process and their quality.

**Figure 2:**
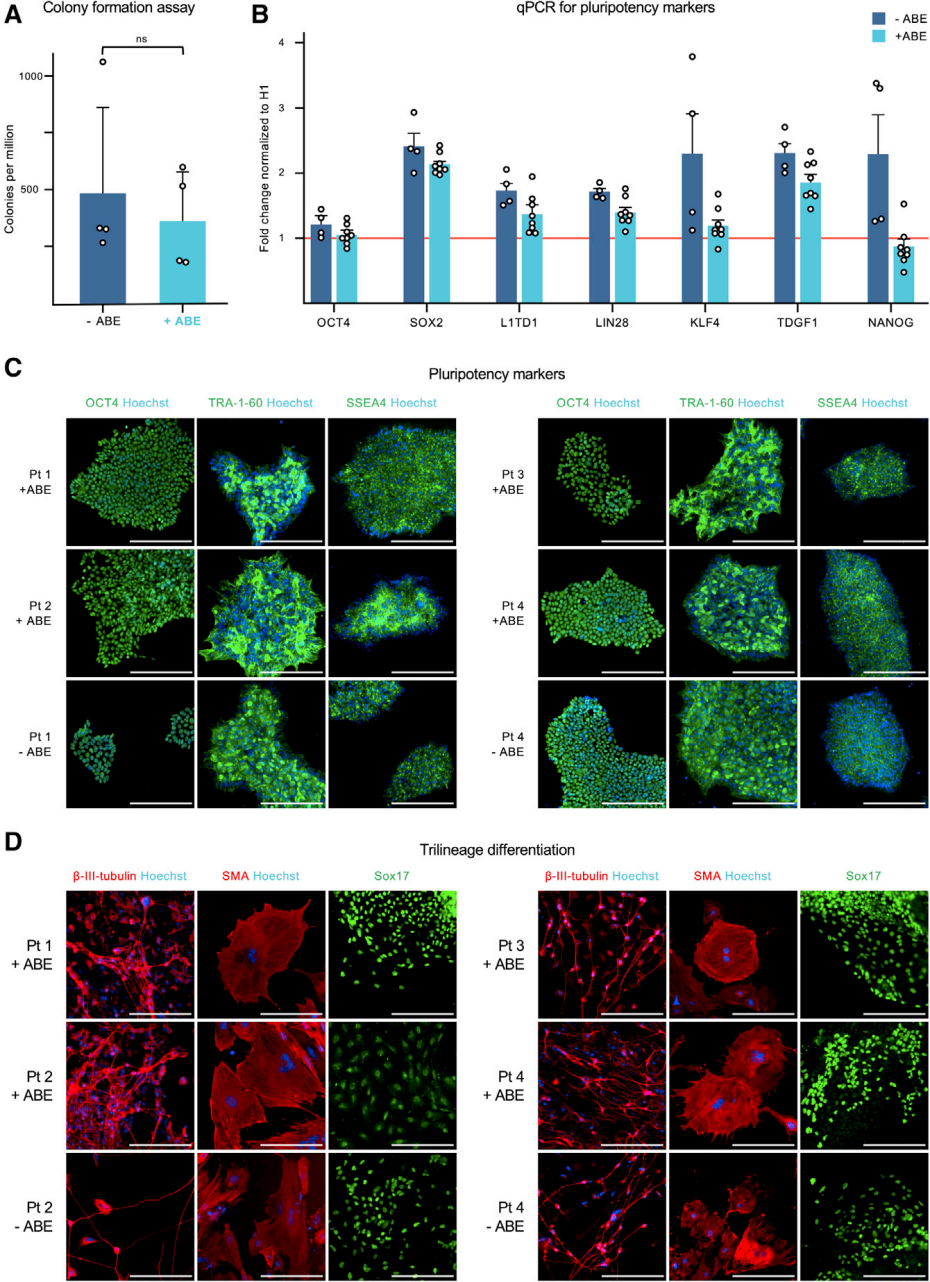
Phenotypic analysis of monoclonal hiPSCs derived from simultaneously edited and reprogrammed primary fibroblasts (A) Colony formation assay after the electroporation of 1 million fibroblasts with the three reprogramming vectors in the presence (+ABE, n = 4) or absence (−ABE, n = 4) of the ABEmax RNA construct and the sgRNA. Each point represents an independent assay from each of the four patient-derived fibroblast populations. Data are represented as mean + standard deviation. (B) qPCR results for the expression levels of OCT4, SOX2, L1TD1, LIN28, KLF4, TDGF1, and NANOG in one non-ABE (−ABE, n = 4) and two base-edited (+ABE, n = 8) hiPSC lines per patient. The eight independently edited hiPSC lines were obtained through reprogramming plus simultaneous adenine base editing. The expression levels were normalized to those of the commercial H1 human embryonic stem cells, illustrated by the orange line. Each point represents an independent hiPSC line. Data are represented as mean + SEM. There were no significant differences between −ABE and +ABE. (C) Immunofluorescence staining of four representative hiPSC lines base edited at the targeted adenine (+ABE) and their non-ABE controls (−ABE) for pluripotency markers OCT4, TRA-1-60, and SSAE4. (D) Immunofluorescence staining of embryoid bodies derived from representative hiPSC lines base edited at the targeted adenine (+ABE) and their non-ABE controls (−ABE). β-III-Tubulin (ectoderm), smooth muscle actin (SMA) (mesoderm), and Sox17 (endoderm). *NOTCH3*: patient 1 and patient 2. *LDLR*: patient 3 and patient 4. Hoechst, in blue, is a nuclear marker. The white bar represents 200 μm.

First, we evaluated the reprogramming efficiency of our method. In the colony formation assay performed with the four independent fibroblast populations, we did not observe significant differences with the inclusion or not of the ABEmax RNA construct in the electroporation along with the episomal reprogramming vectors. In every case, the number of colonies per million fibroblasts remained above 170 (Figure 2A).

Second, to assess the pluripotency of the hiPSCs generated, we compared two on-target edited lines from each of the four patients with their corresponding controls not treated with the ABEmax construct. All hiPSC lines exhibited the cellular morphology characteristics of hESCs: large nuclei and scant cytoplasm (International Stem Cell Initiative et al., 2007). The hiPSC lines reprogrammed along or without the ABEmax RNA construct did not significantly differ in the expression of seven essential pluripotency markers: OCT4, SOX2, L1TD1, LIN28, KLF4, TDGF1, and NANOG (International Stem Cell Initiative et al., 2007) (Figure 2B). The hiPSC expression of each marker was similar or superior to that of the H1 hESCs. The eight independently edited hiPSC lines and their corresponding four controls showed similar positive immunofluorescence staining for the markers OCT4, TRA-1-60, and SSEA-4 (Figures 2C and S1B).

Finally, to assess the pluripotency potential, we performed a trilineage differentiation test with the eight edited lines and their corresponding controls. Positive immunostainings of characteristic markers suggest successful differentiation into ectoderm (β-III-tubulin), mesoderm (smooth muscle actin [SMA]), and endoderm (SOX17) (Figures 2D and S1C). Altogether, these results confirmed pluripotency of the generated hiPSC lines.

### RNA-based ABEmax editing is genetically safe and robust

**Figure 3:**
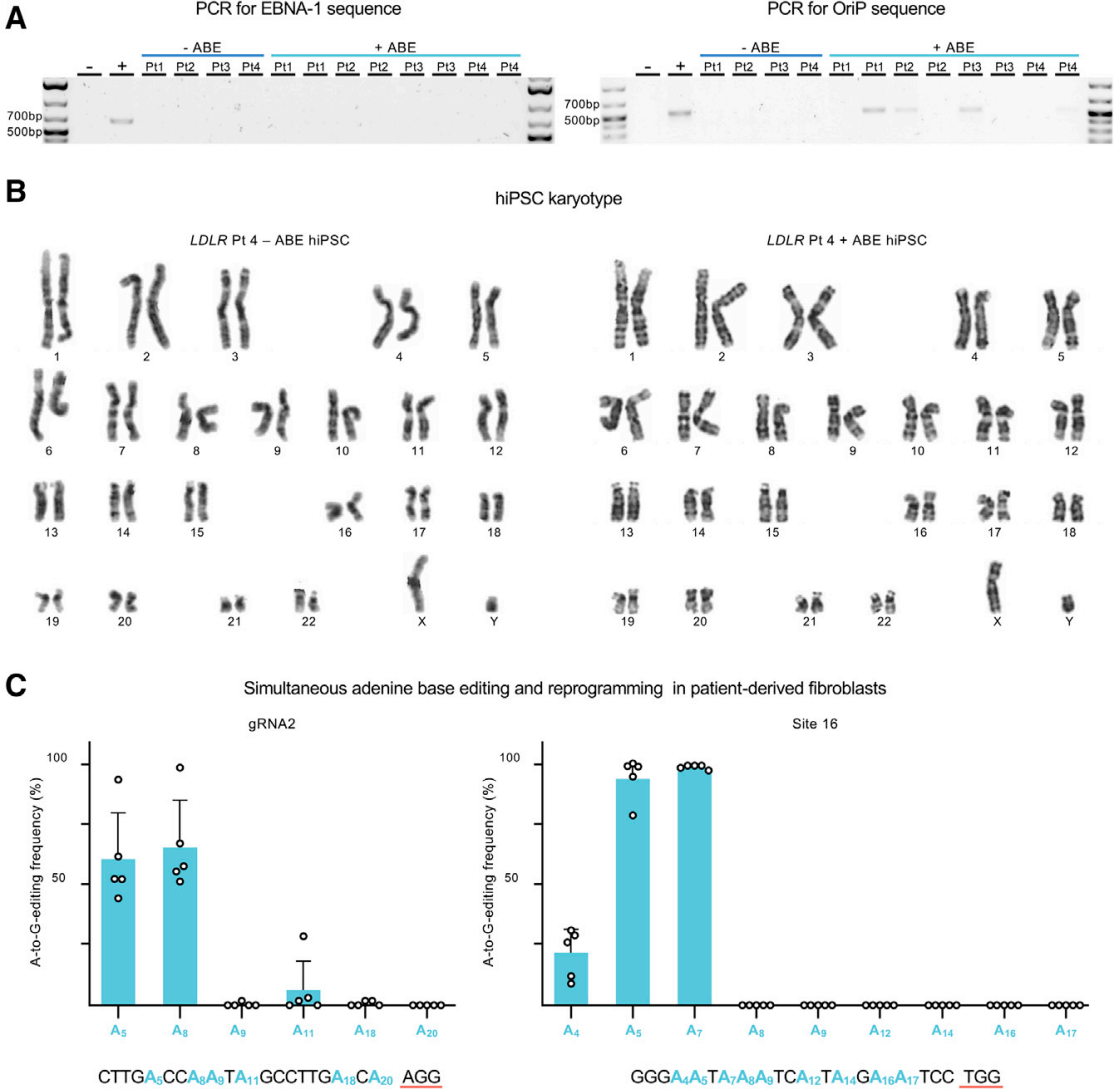
Genetic safety and robustness of base editing and simultaneous hiPSC generation (A) PCR to detect episomal vector retention into four hiPSC lines reprogrammed in absence of the ABEmax construct (−ABE) and eight independently base-edited hiPSC lines (+ABE). Two different plasmid regions are targeted in these PCRs: EBNA-1 and OriP. Water control (−), positive control (+), *NOTCH3*: patient 1 (Pt 1) and patient 2 (Pt 2). *LDLR*: patient 3 (Pt 3) and patient 4 (Pt 4). (B) Karyotyping of a representative base-edited hiPSC lines and its non-ABE control. (C) Bar graphs depicting the A-to-G editing frequency on the targeted loci: gRNA2 (chr11:-5254881) and Site 16 (chr1:-179826685). Each point represents an independent event of simultaneous reprogramming and base editing on primary fibroblasts derived from five different donors (n = 5). Data are represented as mean + standard deviation.

The ABEmax RNA and sgRNA molecules rarely integrate into the genome. However, for the reprogramming of primary fibroblasts, we employed three episomal vectors that could randomly integrate into the cell genome. The reported retention of episomal vector sequences stands at approximately 30% (Okita et al., 2011; Schlaeger et al., 2015). As the ABEmax creates a single-strand break (SSB) in the targeted sequence, the risk of episomal vector integration is theoretically higher when performing the base editing and the reprogramming simultaneously. To assess the vector sequence retention, we ran a sensitive PCR against two different regions, OriP and Epstein-Barr nuclear antigen-1 (EBNA-1), shared by the three episomal vectors employed for the reprogramming (Yu et al., 2009) (Figure 3A). From the eight corrected colonies and their corresponding four non-ABE controls, we detected the OriP region present in four of the edited lines, whereas the EBNA-1 band was absent.

To assess the chromosomal integrity of the ABEmax-edited hiPSC lines, we examined the karyotype of eight edited lines and their corresponding controls (Figure 3B). The 12 hiPSC lines showed a normal karyotype. The hiPSC generation process based on episomal EBNA-1 plasmids for transgenic expression of reprogramming factors may result in about 11% aneuploid hiPSC lines (Schlaeger et al., 2015); in this study, however, we did not observe any abnormalities.

ABEmax has proven even safer and more specific than the canonical SpCas9 (Liang et al., 2019; Zuo et al., 2019). However, with the edit of a genomic region of interest, similar sequences in the genome could also unintentionally mutate. To address this risk, we evaluated the off-target activity in three edited hiPSC lines per patient and their corresponding non-ABE controls. First, we retrieved the top 10 off-target regions predicted by two different software systems: IDT (Integrated DNA Technologies, Redwood City, CA) and CRISPOR (Concordet and Haeussler, 2018). Then, we selected the six more likely off-target regions associated with the *NOTCH3* sgRNA and four for the *LDLR* sgRNA (Table 1). Sanger sequencing results confirmed the absence of off-target edits in all of the analyzed regions.

**Table 1:** Off-target analysis for the single guides employed to correct the *NOTCH3* and *LDLR* mutations

	Software for prediction	Sequence	PAM	#MM	Gene	Locus	Editing
*NOTCH3*							

On-target		CAGCAGGCACCGTGGGCACA	AAG	0	exon: *NOTCH3*	chr19: +15192237	positive
Off-target	IDT	GTGCAGG-ACCGTGGGCACA	GAG	3	intergenic	chr13: −99980088	negative
Off-target	IDT	ATGCAGGGGCCGTGGGCACA	GAG	4	intergenic	chr18: −49569320	negative
Off-target	IDT	CTGCAGGGACAGTGGGCACA	AGG	3	exon: *EVA1A*	chr2: −75493164	negative
Off-target	CRISPOR	TAGCAGGCATAGTGGACACA	AGG	4	intron:C10orf67	chr10: +23578980	negative
Off-target	CRISPOR	TAGTGGGCACCGTGGACACA	AGG	4	intergenic	chr3: +13922911	negative
Off-target	CRISPOR	CAGCACGTACCATGGGCACA	AGG	3	exon: *MYRF*	chr11: −61548252	negative

*LDLR*							

On-target		CAACCAGAAGACCATCTTGG	AGG	0	exon: *LDLR*	chr19: +11116931	positive
Off-target	IDT	CATCCAG-GGACCATCTTGG	CAG	3	intergenic	chr20: +49698697	negative
Off-target	IDT	CATCTAG-GGACCATCTTGG	CAG	4	intergenic	chr20: +49701517	negative
Off-target	CRISPOR	TTACCAGAAGACCATCTTGT	AGG	3	intron: *SCFD1*	chr14: +30726353	negative
Off-target	CRISPOR	AATCCAAAAGACCATCTTAG	AGG	4	intergenic	chr4: −44433731	negative

The *in silico* predicted off-target regions that were sequenced to check unwanted ABEmax-induced A⋅T to G⋅C mutations are shown. The bases in red are the mismatches between each off-target sequence and the original target. #MM, number of mismatches.

Finally, to further test the robustness of our method for simultaneous reprogramming and base editing, we applied our approach to fibroblasts derived from five different donors. We independently targeted two non-related loci using previously described sgRNAs: gRNA2 (chr11:-5254881) (Traxler et al., 2016) and Site16 (chr1:-179826685) (Gaudelli et al., 2017). We collected all cells 20 days after electroporating the primary fibroblasts with the reprogramming vectors, the ABEmax, and either of the sgRNAs. Each of these 10 bulk population samples, one per donor and sgRNA, contained the DNA of at least 6 million cells. In every case, the Sanger sequencing revealed a remarkable biallelic on-target editing efficiency within a well-defined window of editing (Figure 3C). The absolute biallelic A-to-G editing frequency on the locus gRNA2 was 60.8 ± 17.6 and 65.6 ± 17 for the bases A5 and A8, respectively. On the locus Site 16, the editing was 94.4 ± 7.9 and 98.4 ± 0.8 for bases A5 and A7, respectively.

### Correcting the FH-Pogosta point mutation restores the LDLR activity

**Figure 4:**
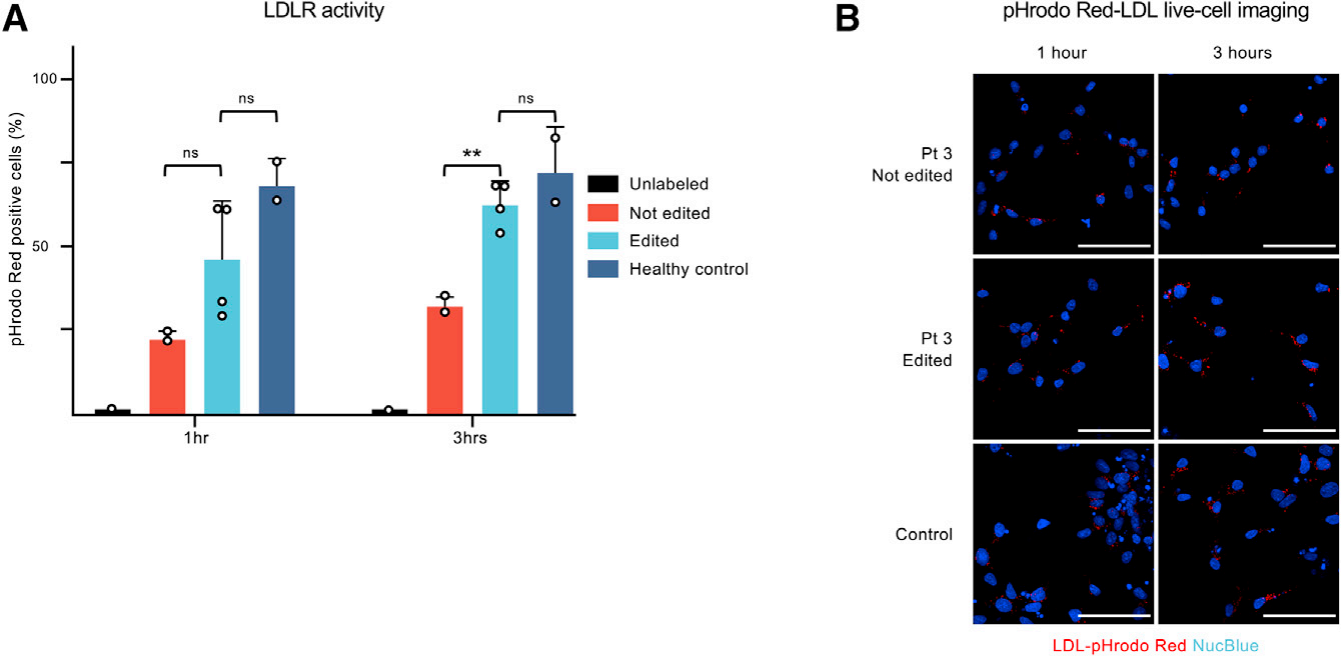
LDLR activity restoration upon gene correction in definitive endoderm (A) Receptor-mediated endocytosis of labeled human low-density lipoprotein (LDL) in hiPSC-derived endoderm. The y axis represents the percentage of pHrodo Red-positive cells measured by flow cytometry after an incubation of 1 or 3 h. Unlabeled (n = 1), not edited (hiPSCs from Pt 3 and Pt 4; n = 2), edited (two independent hiPSC lines per patient, Pt 3 and Pt 4; n = 4), healthy control (two independent hiPSC lines; n = 2). Data are represented as mean + standard deviation. ^∗∗^p = 0.004. (B) Live-cell imaging illustrating LDL-pHrodo Red endocytosis. The white bar represents 100 μM.

Having generated and validated gene-edited patient-derived hiPSC lines, we wanted to assess the phenotype restoration on the corrected FH-Pogosta lines. We employed one not-edited and two edited hiPSC lines per patient (Pts 3 and 4) and two lines from donors without this mutation (Pts 1 and 2). To test the activity of the low-density lipoprotein (LDL) receptor (LDLR), we first differentiated these eight independent hiPSC lines into definitive endoderm. On day 7, we incubated the cells for 1 or 3 h with human LDL labeled with pHrodo Red. This fluorophore activates only at the low pH inside the lysosomes. We expected less efficient receptor-mediated LDL endocytosis in the lines carrying the pathogenic mutation in LDLR than in the healthy donors. Then, we hypothesized that the correction of the FH-Pogosta mutation would restore the LDLR activity. We analyzed each population by flow cytometry and confirmed a significant increase in the percentage of pHrodo Red-positive cells in the gene-edited population (Figure 4A). To illustrate the pHrodo Red-labeled LDL endocytosis, we imaged living cells from the not-edited, edited, and control populations (Figure 4B).

## Discussion

Here we have developed and tested a highly efficient method combining base editing and simultaneous hiPSC generation. Starting from a patient biopsy, this approach requires just a single step of monoclonal expansion. In contrast, performing reprogramming and gene editing as separate steps requires two monoclonal expansions, one to obtain a monoclonal hiPSC line and a second after the genetic edition. These costly sequential expansion steps lengthen the process to more than 4 months, while further increasing the risk of spontaneous mutations or chromosomal abnormalities.

It has been shown previously that simultaneous gene editing and reprogramming effectively reduces the cell culture time and skips one expansion step. However, previous techniques relied on Cas9-induced DSBs to stimulate either NHEJ or HDR, with editing efficiencies ranging from 2% to 45% (Howden et al., 2015; Howden et al., 2018; Kim et al., 2016; Tidball et al., 2017, 2018; Wen et al., 2018). We describe here a much more efficient method (96% without requiring any form of selection), which is also DSB-free, reducing the risk of DNA damage. Another considerable advantage of our approach is that the delivery system for the gene-editing tool is completely RNA based. These attributes minimize cell toxicity and off-target effects.

We have chosen to generate patient-derived hiPSCs by episomal reprogramming vectors. This system is state of the art, but it increases the likelihood of hiPSC quality variability and could cause plasmid integrations into the genome or aneuploidies (Schlaeger et al., 2015). More advanced but expensive reprogramming technologies employ safer vectors for delivery, such as Sendai RNA virus (Fusaki et al., 2009; Nishimura et al., 2017) and RNA vectors (Warren et al., 2010). Given that our mRNA-like ABEmax construct performs the desired gene editing rapidly and efficiently in a variety of cell types, such as HEK293, fibroblasts, and hiPSCs, it could also work together with these alternative reprogramming technologies. In this scenario, the patient's primary cells would undergo simultaneous editing and reprogramming using only RNA tools, which may help to increase the hiPSC quality while minimizing the risk of genetic alterations.

This method significantly simplifies and enhances the work of researchers and biobanks, providing a cleaner and more efficient way to convert A⋅T base pairs to G⋅C base pairs, which could potentially correct a large portion of human pathogenic SNPs (Gaudelli et al., 2017) or even enable targeted exon skipping (Winter et al., 2019). Considering the several CRISPR-based technologies available (Anzalone et al., 2020) and the rapid advance of gene therapy toward clinics (Maldonado et al., 2020), we envision a near future where all pathogenic SNPs are efficiently base edited, DSB-free, and without PAM limitations. Similarly, hiPSC research also dynamically evolves, getting closer to delivering cell and tissue therapy applications. Our approach has the potential to improve the connection between these two fields. Replacing the ABEmax sequence in the IVT plasmid presented in this article allows the transcription of virtually any CRISPR-based technology into an efficient mRNA-like construct.

From a therapeutic perspective, the robustness and swiftness of this method would significantly improve the generation of disease-corrected hiPSCs for therapies based on autologous cell transplantation. Furthermore, our mRNA-like construct could serve to convert A⋅T base pairs to G⋅C base pairs in primary cells, such as skin stem cells, which are good candidates for autologous transplantation (Hirsch et al., 2017). RNA delivery of gene-editing tools could efficiently correct pathogenic mutations in this kind of cell while avoiding the risks derived from viral integration. *In vivo* delivery of our mRNA-like construct through lipid nanoparticles (Witzigmann et al., 2020) would also broaden its range of applications. Furthermore, the sgRNAs presented in this article are strong candidates to develop gene-editing therapies to correct the mentioned point mutations causing CADASIL and FH-Pogosta.

From a research perspective, our method allows the rapid generation of *in vitro* models, where several independently gene-edited hiPSC lines would help to discern meaningful results from line and differentiation-related variability. In this article, we exemplified this point by assessing the restoration of the LDLR activity upon gene correction. This platform would serve, for instance, to test drugs and small molecules aiming to enhance the receptor-mediated LDL endocytosis in FH-Pogosta patient-derived liver organoids.

The method presented here possesses valuable characteristics to improve the gene editing of hiPSCs. Its remarkably high editing efficiency reduces the workload to find and validate colonies with the desired genotype, which is a constraint in HDR protocols. Furthermore, this robust and simple approach does not need any extra plasmid cloning, cell selection, or single-cell sorting steps. The transient, rapid, and high expression of our RNA construct allows for biallelic or multiplexed base editing. Starting from primary cultures of patient-derived fibroblasts, our approach yielded tens of on-target edited hiPSC lines in less than 6 weeks, without compromising their genetic integrity nor their pluripotent functionality. Given the fundamental importance of patient-derived isogenic hiPSC lines for research and the possibility for future therapies, we believe that many other researchers may benefit from our optimized workflow.

## Experimental procedures

### *In vitro* transcription ABEmax, BE3, and BE4 plasmid construction

pCMV_ABEmax was a gift from David Liu (Addgene plasmid 112095; http://n2t.net/addgene:112095; RRID: Addgene_112095). The open reading frame (ORF) was amplified through PCR from the start codon to the last codon of the nuclear localization signal (NLS), where we added a stop codon. The forward primer had an XmaI restriction site upstream of the start codon (Table S1), and the reverse primer contained a NotI restriction site downstream of the stop codon. These restriction enzymes served to clone the 5,412 bp PCR product into the IVT backbone (Torres-Padilla et al., 2007) without altering the original ABEmax sequence.

We employed the same approach to clone the C⋅G to T⋅A base editors BE3 and BE4 (Komor et al., 2016, 2017) into the IVT backbone. The forward primer had a BamHI restriction site upstream of the start codon instead of XmaI (Table S1). BE3 and BE4 were a gift from David Liu (Addgene plasmid 73021; http://n2t.net/addgene:73021; RRID: Addgene_73021) and (Addgene plasmid 100802; http://n2t.net/addgene:100802; RRID: Addgene_100802).

Our plasmids for ABEmax, BE3, and BE4 IVT were deposited in Addgene (plasmids 171761, 177015, and 171762, respectively).

### Plasmids for transgenic expression of reprogramming factors

For transgenic reprogramming we employed pCXLE-hSK (Addgene, 27078; Okita et al., 2011), pCXLE-hUL (Addgene, 27080; Okita et al., 2011), and pCXLE-hOCT3/4 (Addgene, 27077; Okita et al., 2011).

### ABEmax RNA *in vitro* transcription

Employing the ABEmax IVT plasmid as a DNA template, we followed the manufacturer protocol for T3 RNA transcription (mMESSAGE mMACHINE T3 Transcription Kit, Thermo Fisher Scientific, Invitrogen, cat. no. AM1348). The plasmid was linearized by SfiI restriction (Thermo Fisher Scientific, cat. no. FD1824).

### Ethical permit

The patient skin biopsies and clinical data research were approved by the ethical committee of Helsinki and Uusimaa Hospital region (diary no. HUS/2754/2019).

### Fibroblast culture from skin biopsy

A medical doctor took the skin biopsy from the ventral side of the lower arm of the donor applying local anesthesia. The sample was collected in a 15 ml tube with 5 mL DMEM (Sigma, 6546, fetal bovine serum [FBS]) supplemented with 20% FBS (Life Technologies, 10106-169), 1% GlutaMAX (Life Technologies, 35050-038), and 1% penicillin-streptomycin (Life Technologies, 15140-122). The skin sample was manually disaggregated into smaller pieces, which were seeded in a 60 mm plate and covered with sterile glass coverslips (10 mm diameter). The biopsy was kept in culture in the previously described medium until the fibroblasts formed a confluent monolayer and were harvested for further expansion.

### Fibroblast electroporation for hiPSC induction

We electroporated 1 million fibroblasts in a final volume of 100 μL R buffer containing 1.5 μg of each of the three reprogramming plasmids plus 2 μL of electroporation enhancer from IDT. The electroporation settings were three pulses, pulse width of 10 ms, and 1650 V. Cells were seeded in a 60 mm Matrigel-coated plate with mouse embryonic fibroblast (MEF) medium.

### Fibroblast electroporation for simultaneous base editing and hiPSC induction

To the electroporation solution detailed above, we added 23 μg of ABEmax RNA and 10 μg of sgRNA. The electroporation settings remained the same.

### hiPSC electroporation for base editing

We electroporated 1 million hiPSCs in a final volume of 100 μL R buffer containing 23 μg of ABEmax RNA, 10 μg of sgRNA, and 2 μL of electroporation enhancer. The electroporation settings were two pulses, pulse width of 20 ms, and 1100 V. Cells were seeded in a 35 mm Matrigel-coated plate with Essential 8 (E8) medium (Thermo Fisher Scientific, A1517001) containing 10 μM ROCK inhibitor, 0.1% CloneR (STEMCELL Technologies, 05888), and 175 ng/mL recombinant B18R protein (STEMCELL Technologies, 78075). At 24 h after electroporation, the medium was changed to E8 plus B18R.

### Electroporation equipment

The Neon Transfection System 100 μL Kit (Thermo Fisher Scientific, MPK10096) was used for electroporation.

### Colony assay

One million fibroblasts were electroporated with the three reprogramming plasmids, as previously described, including or skipping the ABEmax RNA and the sgRNA. The cells were then seeded in a 100 mm Matrigel-coated plate (Corning, 356231) with MEF medium (10% FBS and 1% GlutaMAX). Four days after electroporation, the medium was changed to 50% MEF and 50% hES (DMEM/F1220; Life Technologies, 31331-028; supplemented with 20% KnockOut Serum Replacement, Life Technologies, 10828-028; 0.0915 mM 2-mercaptoethanol, Life Technologies, 31350-010; 1% non-essential amino acids, Life Technologies, 11140-035; and 6 ng/mL bFGF, Sigma, F0291) plus 0.25 mM sodium butyrate. Fifteen days after electroporation, the cells were fixed for 10 min in paraformaldehyde (PFA) 4%, washed with PBS, and then stained with NBT/BCIP (Roche)-containing buffer (0.1 M Tris HCl [pH 9.5], 0.1 M NaCl, 0.05 M MgCl_2_) until a purple precipitate formed. The reaction was stopped by PBS wash.

### hiPSC culture and clonal isolation

Five days after the primary fibroblasts were electroporated with the reprogramming factors, they were transferred into six wells of a Matrigel-coated six-well plate in decreasing concentrations (40%, 35%, 25%, 15%, 10%, and 5% of the total amount of cells). From this point, the medium was changed to hES supplemented with 0.25 mM sodium butyrate (Sigma-Aldrich). Twelve days after electroporation, the medium was changed to E8 medium. From day 11 to day 17 after electroporation, the visible hiPSC monoclonal colonies were picked from the fibroblast monolayer employing a 100 μL tip and transferred to a Matrigel-coated 24-well plate with E8 medium containing 10 μM ROCK inhibitor.

### Embryoid body generation

For the embryoid body assay, hiPSCs were passaged as small clumps on ultra-low attachment six-well culture plates in hES medium without bFGF. The medium was supplemented with 10 μM ROCK inhibitor for the first 24 h. The PSCs were cultured as aggregates for 2 weeks and the medium was changed every other day. After 2 weeks, the embryoid bodies were plated onto a 24-well plate and cultured for 7 days in hES medium without bFGF. Thereafter, the embryoid bodies were fixed with 4% PFA for 30 min for immunocytochemistry.

### hiPSC differentiation into definitive endoderm

We followed a 7 day differentiation protocol in monolayer, as previously described (Zabulica et al., 2021).

### LDLR activity assay

Fifty thousand hiPSC-derived definitive endothelial cells were resuspended and plated in a well of a Matrigel-coated 24-well culture plate. On the following day, the cells were incubated for 1 or 3 h in 300 μL of RPMI 1640 medium + GlutaMAX (Gibco, 61870-010) plus 3 μL of LDL from human plasma pHrodo Red (Thermo Fisher Scientific, L34356). After the incubation, cells were dissociated for 5 min with StemPro Accutase cell dissociation reagent (Thermo Scientific, A1110501), resuspended in PBS-FBS 5%, and centrifuged for 5 min at 200*g*. The cell pellet was resuspended in 300 μL of PBS-FBS 5% and quickly processed by flow cytometry.

### Live-cell imaging for pHrodo Red-labeled LDL

Two hundred thousand hiPSC-derived definitive endothelial cells were resuspended and plated in a Matrigel-coated 35 mm plate with a glass bottom. On the following day, the cells were incubated for 1 or 3 h in 300 μL of RPMI 1640 medium + GlutaMAX plus 3 μL of LDL from human plasma pHrodo Red and 10 μL of NucBlue (Thermo Scientific, R37605). After the incubation and without changing their medium, cells were imaged on a Zeiss Axio Observer Z1 with Apotome at 20× magnification. All plates were equally treated and imaged with the same microscope parameters; images were processed with ZEN2 software.

### Karyotype analysis

Samples were prepared for karyotyping using a protocol adapted from Howe et al. (2014); 2.0 × 10^6^ cells were suspended in medium supplemented with 0.1 μg/mL KaryoMAX colcemid solution in PBS and incubated for 4 h at 37°C. Cells were resuspended in 0.075 M KCl and incubated at 37°C for 10 min. Fixative (3:1 ratio of methanol and acetic acid) was added dropwise to the cell suspension. Fixation was repeated three times before storing the samples at 4°C until shipping. Karyotyping was performed as a service by Ambar in Barcelona, Spain.

### Immunostaining

The cells were plated onto 24-well culture plates before the immunostainings. Cells were fixed with 4% PFA (Fisher Chemical) in PBS for 15 min. The cells were then permeabilized by 0.5% Triton X-100 in PBS for 10 min and treated with Ultravision blocker (Thermo Scientific) for 10 min. Primary antibodies were diluted in 0.1% Tween in PBS, added to the wells, and incubated for 24 h in dark at 4°C on a Stuart SSL4 seesaw rocker. Secondary antibodies, and Hoechst 33342 (Thermo Fisher Scientific) to stain the nuclei, were diluted in 0.1% Tween in PBS and added to the wells. The wells were then incubated in the dark at room temperature (RT) for 30 min on the seesaw rocker.

The primary antibodies used in this study were OCT4 (1:500 goat, Santa Cruz, sc-8628), TRA-1-60 (1:500 mouse, Thermo Fisher Scientific, MA1-023), SSEA (1:1,000 mouse, Millipore, MAB4304), SOX17 (1:500 goat, R&D Systems, AF1924), α-SMA (1:500 mouse, Sigma, A2547), and β-tubulin III (1:500 rabbit, Abcam, Ab18207).

The secondary antibodies used were Alexa Fluor 488 anti-goat (1:500 donkey, Invitrogen, A11055), Alexa Fluor 488 anti-mouse (1:500 donkey, Invitrogen, A21202) and anti-rabbit (1:500 donkey, Invitrogen, A21206), and Alexa Fluor 594 anti-mouse (1:500 donkey, Invitrogen, A21203) and anti-rabbit (1:500 donkey, Invitrogen, A21207).

## Author contributions

Conceptualization, S.J.; methodology, S.J.; investigation, S.J., T.K., and J.S.; formal analysis, S.J., R.M., and J.S.; visualization, S.J., T.K., R.M., and J.S.; writing – original draft, S.J.; writing – review & editing, all of the authors contributed; resources, T.O. and K.W.; supervision, K.W. and R.T.; project administration, S.J. and K.W.; funding acquisition, K.W. and T.O.

## Conflict of interests

The authors declare no competing interests.

## Supplemental information

Document S1. Figure S1 and Table S1

Document S2. Article plus supplemental information
