# Supplementary material for: Simultaneous high-efficiency base editing and reprogramming of patient fibroblasts
Source: Stem Cell Reports. 2021 Nov 24;16(12):3064–75. doi: 10.1016/j.stemcr.2021.10.017 (PMC8693657; doi:10.1016/j.stemcr.2021.10.017)
Supplement: Document S1. Figure S1 and Table S1 [file mmc1.pdf]

Stem Cell Reports, Volume *16*

Supplemental Information

Simultaneous high-efficiency base editing and reprogramming of patient fibroblasts

Sami Jalil, Timo Keskinen, Rocío Maldonado, Joonas Sokka, Ras Trokovic, Timo Otonkoski, and Kirmo Wartiovaara

Figure S1: **Direct adenine base editing in patient-derived hiPSC and supplemental immunofluorescence staining image**

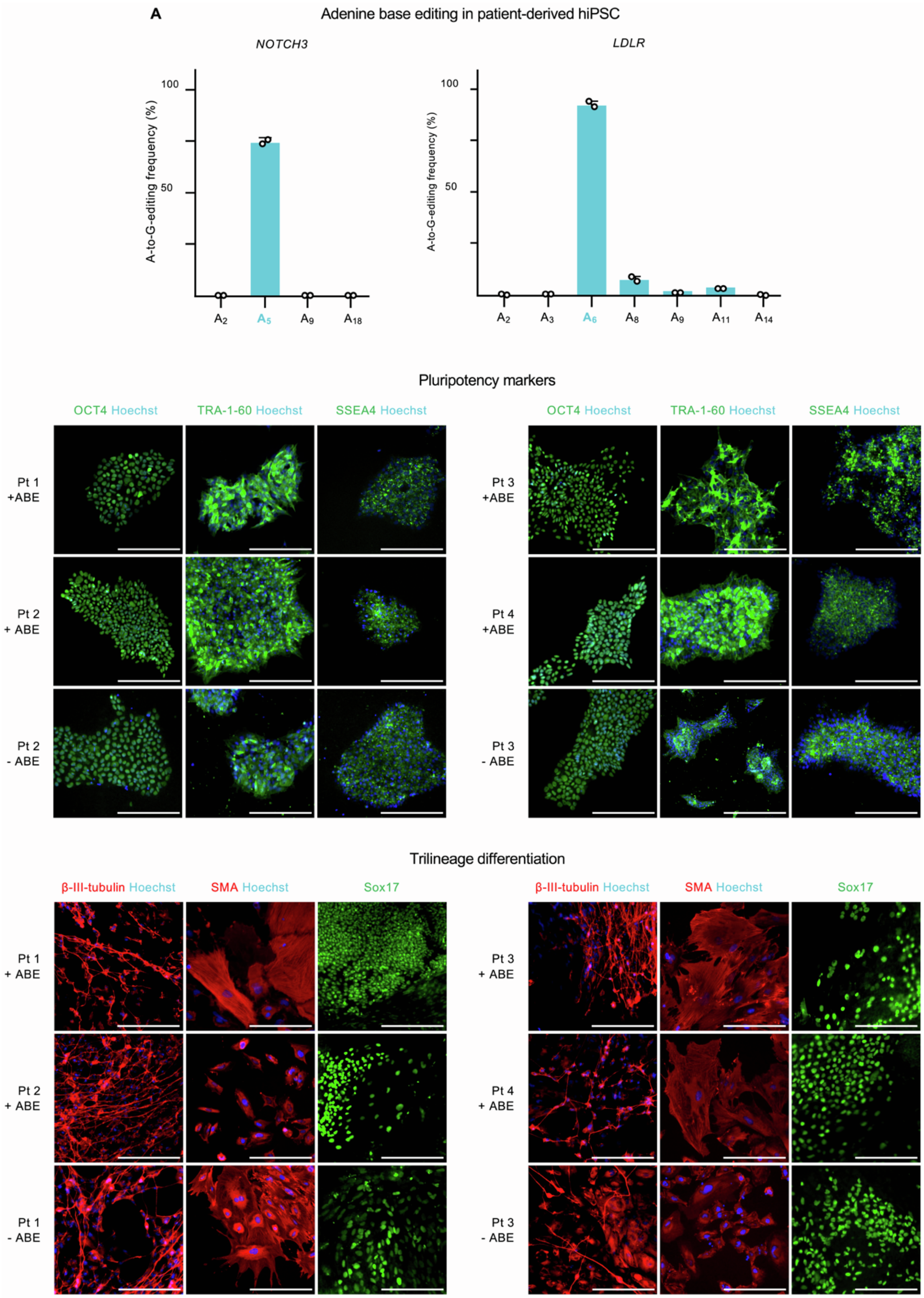

(A) One non-ABE hiPSC line per patient (two for NOTCH3 and two for LDLR) were electroporated with the ABEmax RNA construct and the corresponding sgRNA. After seven days, each hiPSC bulk population was sequenced. The bar graphs depict the average editing efficiency on each adenine in the loci. Data are represented as mean + standard deviation.

(B) Immunofluorescence staining for pluripotency markers OCT4, TRA-1-60, and SSAE4 of the remaining four clones base-edited at the targeted adenine (+ ABE) and their non-ABE controls (- ABE) not shown in Figure 2C.

(C) Immunofluorescence staining of embryoid bodies derived from the remaining clones base-edited at the targeted adenine (+ ABE) and their non-ABE controls (- ABE) not shown in Figure 2D. β-III-tubulin (ectoderm), smooth muscle actin (SMA) (mesoderm), and Sox17 (endoderm). NOTCH3: patient 1 and patient 2. LDLR: patient 3 and patient 4. Hoechst, in blue, is a nuclear marker. The white bar represents 200µm.

Table S1: Single guide RNA and primers employed in this study

Name	Sequence	Function	Comments	Vendor
**Single guide RNA**				
sg_*NOTCH3*	mC*mA*mG*rCrArGrGrCrArCrCrGrUrGrGrGrCrArCrArGrUrUrUrArGrArGrCrUrArGrArArArUrArGrCrArArGrUrUrArArArArUrArArGrGrCrUrArGrUrCrCrGrUrUrArUrCrArArCrUrUrGrArArArArArGrUrGrGrCrArCrCrGrArGrUrCrGrGrUrGrCmU*mU*mU*rU	Target the c.475C>T mutation in *NOTCH3*.	RNA oligo	IDT
sg_*LDLR*	mC*mA*mA*rCrCrArGrArArGrArCrCrArUrCrUrUrGrGrGrUrUrUrUrArGrArGrCrUrArGrArArArUrArGrCrArArGrUrUrArArArArUrArArGrGrCrUrArGrUrCrCrGrUrUrArUrCrArArCrUrUrGrArArArArArGrUrGrGrCrArCrCrGrArGrUrCrGrGrUrGrCmU*mU*mU*rU	Target the c.1784G>A mutation in *LDLR*	RNA oligo	IDT
sg_gRNA2	mC*mT*mT*rGrArCrCrArArTrArGrCrCrTrTrGrArCrArGrUrUrUrUrArGrArGrCrUrArGrArArArUrArGrCrArArGrUrUrArArArArUrArArGrGrCrUrArGrUrCrCrGrUrUrArUrCrArArCrUrUrGrArArArArArGrUrGrGrCrArCrCrGrArGrUrCrGrGrUrGrCmU*mU*mU*rU	Target the gRNA locus in chromosome 11	RNA oligo	IDT
sg_Site 16	mG*mG*mG*rArArTrArArArTrCrArTrArGrArArTrCrCrGrUrUrUrUrArGrArGrCrUrArGrArArArUrArGrCrArArGrUrUrArArArArUrArArGrGrCrUrArGrUrCrCrGrUrUrArUrCrArArCrUrUrGrArArArArArGrUrGrGrCrArCrCrGrArGrUrCrGrGrUrGrCmU*mU*mU*rU	Target the site 16 locus in chromosome 1	RNA oligo	IDT
**PCR primers**				
*NOTCH3*_FW	TCTTTGTGTCTGGGGCCATC	Amplify the region containing the pathogenic mutation	DNA oligo	Sigma
*NOTCH3*_RV	GACAGTCGTCCACGTTCACT	Amplify the region containing the pathogenic mutation	DNA oligo	Sigma
1st_IDT_off-target_ *NOTCH3*_FW	ACTTCTCAGCCCAAGGATGC	Amplify the region containing a possible off-target	DNA oligo	Sigma
1st_IDT_off-target_ *NOTCH3*_RV	CGCTCCAGTCTTTCTGCGTA	Amplify the region containing a possible off-target	DNA oligo	Sigma
2nd_IDT_off-target_ *NOTCH3*_FW	GTTTCCGAGCCATGGGGTTA	Amplify the region containing a possible off-target	DNA oligo	Sigma
2nd_IDT_off-target_ *NOTCH3*_RV	TTGGGAGCATTCCGGCTTAG	Amplify the region containing a possible off-target	DNA oligo	Sigma
3rd_IDT_off-target_ *NOTCH3*_FW	AGTCGCGTTTCTCCGATCTC	Amplify the region containing a possible off-target	DNA oligo	Sigma
3rd_IDT_off-target_ *NOTCH3*_RV	GAGGATGGCAGTGAGGACAC	Amplify the region containing a possible off-target	DNA oligo	Sigma
1st_CRISPOR_off-target_*NOTCH3*_FW	GCAGATGGTCAGTGATCCCA	Amplify the region containing a possible off-target	DNA oligo	Sigma
1st_CRISPOR_off-target_*NOTCH3*_RV	ACAGCAAAAATGTCCGTCGC	Amplify the region containing a possible off-target	DNA oligo	Sigma
2nd_CRISPOR_off-target_*NOTCH3*_FW	AGAGTGTTCCTCTCGGGCT	Amplify the region containing a possible off-target	DNA oligo	Sigma
2nd_CRISPOR_off-target_*NOTCH3*_RV	TCAGGCCTCACCTCAAATGC	Amplify the region containing a possible off-target	DNA oligo	Sigma
3rd_CRISPOR_off-target_*NOTCH3*_FW	GTTTAGTGCCCCTCCAGGTA	Amplify the region containing a possible off-target	DNA oligo	Sigma
3rd_CRISPOR_off-target_*NOTCH3*_RV	AGGCCAGAGGCTGTATGAGC	Amplify the region containing a possible off-target	DNA oligo	Sigma
*LDLR*_FW	CATTGGAGAGGGCGTCACAG	Amplify the region containing the pathogenic mutation	DNA oligo	Sigma
*LDLR*_RV	GTCACAACCAGTTTTCTGCGT	Amplify the region containing the pathogenic mutation	DNA oligo	Sigma
1st_IDT_off-target_ *LDLR* _FW	ATCCCACACCAAAGCCAAAAC	Amplify the region containing a possible off-target	DNA oligo	Sigma
1st_IDT_off-target_ *LDLR* _RV	ACAGCTAGTCTTGTGCTCCA	Amplify the region containing a possible off-target	DNA oligo	Sigma
2nd_IDT_off-target_ *LDLR* _FW	AGTACCCACCCCTGCCTTTA	Amplify the region containing a possible off-target	DNA oligo	Sigma
2nd_IDT_off-target_ *LDLR* _RV	GACCCTGAGGCATGACTTTCA	Amplify the region containing a possible off-target	DNA oligo	Sigma
1st_CRISPOR_off-target_ *LDLR* _FW	CACTTACGCATTCCACACGC	Amplify the region containing a possible off-target	DNA oligo	Sigma
1st_CRISPOR_off-target_ *LDLR* _RV	GTATCTTGGCAGAGCGAGCA	Amplify the region containing a possible off-target	DNA oligo	Sigma
2nd_CRISPOR_off-target_ *LDLR* _FW	CCCTTCCTTGAAGAGTTTAGCCT	Amplify the region containing a possible off-target	DNA oligo	Sigma
2nd_CRISPOR_off-target_ *LDLR* _RV	TTGTCTAGGGAAGCCCATGC	Amplify the region containing a possible off-target	DNA oligo	Sigma
gRNA_FW	GACGTGTCCCATCAAAAATCCT	Amplify the locus Chr11: -5254881	DNA oligo	Sigma
gRNA_RV	TTCCCAGGGTTTCTCCTCCA	Amplify the locus Chr11: -5254881	DNA oligo	Sigma
Site16_FW	CAGGAGTAGGGGTGAGGGAA	Amplify the locus Chr1: -179826685	DNA oligo	Sigma
Site16_RV	ATCTGGTTCCTTCAGCTGGC	Amplify the locus Chr1: -179826685	DNA oligo	Sigma
ABEmax_ORF+XmaI_FW	CGCGGGCCCGGGATCCACAGCCACCATGAAACGGACAGCCGACGGAA	Amplify the ABEmax ORF and add an XmaI restriction site before the start codon	DNA oligo	Sigma
ABEmax_ORF+NotI_RV	ATCCGCGGCCGCTCACCTACTTAGACTTTCCTCTTCTTCTTGGGC	Amplify the ABEmax ORF and add a NotI restriction site after the stop codon	DNA oligo	Sigma
BE3/BE4_ORF+BamHI _FW	CGCGGGCCCGGGATCCACAGCCACCATGAGCTCAGAGACTGGCCCAG	Amplify the BE3 and BE4 ORF, and add a BamHI restriction site before the start codon	DNA oligo	Sigma
BE3/BE4_ORF+NotI _RV	ATCCGCGGCCGCTCACCTACTTAGACTTTCCTCTTCTTCTTGGGA	Amplify the BE3 and BE4 ORF, and add a NotI restriction site after the stop codon	DNA oligo	Sigma
EBNA-1_FW	ATCGTCAAAGCTGCACACAG	Detect the EBNA-1 region from the reprogramming vectors in hiPSC	DNA oligo	Sigma
EBNA-1_RV	CCCAGGAGTCCCAGTAGTCA	Detect the EBNA-1 region from the reprogramming vectors in hiPSC	DNA oligo	Sigma
OriP_FW	TTCCACGAGGGTAGTGAACC	Detect the OriP region from the reprogramming vectors in hiPSC	DNA oligo	Sigma
OriP_RV	TCGGGGGTGTTAGAGACAAC	Detect the OriP region from the reprogramming vectors in hiPSC	DNA oligo	Sigma
**qPCR primers**				
qPCR_OCT4_FW	TTGGGCTCGAGAAGGATGTG	qPCR analysis of OCT4 expression	DNA oligo	Sigma
qPCR_OCT4_RV	GTGAAGTGAGGGCTCCCATA	qPCR analysis of OCT4 expression	DNA oligo	Sigma
qPCR_SOX2_FW	GCCCTGCAGTACAACTCCAT	qPCR analysis of SOX2 expression	DNA oligo	Sigma
qPCR_SOX2_RV	TGCCCTGCTGCGAGTAGGA	qPCR analysis of SOX2 expression	DNA oligo	Sigma
qPCR_L1TD1_FW	TCAGAGATGACAGCATTTGGC	qPCR analysis of L1TD1 expression	DNA oligo	Sigma
qPCR_L1TD1_RV	TTCAGGCAGCAGGTTCTGTTTA	qPCR analysis of L1TD1 expression	DNA oligo	Sigma
qPCR_LIN28_FW	AGGAGACAGGTGCTACAACTG	qPCR analysis of LIN28 expression	DNA oligo	Sigma
qPCR_LIN28_RV	TCTTGGGCTGGGGTGGCAG	qPCR analysis of LIN28 expression	DNA oligo	Sigma
qPCR_KLF4_FW	CCGCTCCATTACCAAG	qPCR analysis of KLF4 expression	DNA oligo	Sigma
qPCR_KLF4_RV	CACGATCGTCTTCCCCTCTT	qPCR analysis of KLF4 expression	DNA oligo	Sigma
qPCR_ TDGF1_FW	TCAGAGATGACAGCATTTGGC	qPCR analysis of TDGF1 expression	DNA oligo	Sigma
qPCR_ TDGF1_RV	TTCAGGCAGCAGGTTCTGTTTA	qPCR analysis of TDGF1 expression	DNA oligo	Sigma
qPCR_NANOG_FW	CTCAGCCTCCAGCAGATGC	qPCR analysis of NANOG expression	DNA oligo	Sigma
qPCR_NANOG_RV	TAGATTTCATTCTCTGGTTCTGG	qPCR analysis of NANOG expression	DNA oligo	Sigma
qPCR_ CycloG_FW	TCTTGTCAATGGCCAACAGAG	qPCR analysis of CycloG expression as a reference housekeeping gene	DNA oligo	Sigma
qPCR_ CycloG _RV	GCCCATCTAAATGAGGAGTTG	qPCR analysis of CycloG expression as a reference housekeeping gene	DNA oligo	Sigma

The table S1 shows the single-guide RNAs (sgRNA) employed to correct the *NOTCH3* and *LDLR* pathogenic point mutations; the sgRNA targeting the loci gRNA2 and Site 16; and the primers that served for amplifying on-target and off-target sequences, plasmid cloning, and qPCR.

The sequences are notated in 5'-to-3' orientation. In the "single guide RNA section", the protospacer is underlined, the non-underlined sequence is the canonical CRISPR-Cas9 tracrRNA. "r_" (rA, rC, rG, rU) refers to ribonucleic bases. "m_*" (mC*, mA*, mG*, mU*) refers to phosphorothioated 2'-O-methyl RNA bases.
